# Comparative and clinical impact of targeted next-generation sequencing in pediatric pneumonia diagnosis and treatment

**DOI:** 10.3389/fmicb.2025.1590792

**Published:** 2025-06-25

**Authors:** Hui Shang, Sini Zou, Zhanying Ma, Qianyu Liang, Ye Zhong, Ling Li, Qishan Chen

**Affiliations:** Department of Pediatrics, Dongguan Maternal and Child Health Care Hospital, Dongguan, China

**Keywords:** pediatric pneumonia, targeted next-generation sequencing (tNGS), bronchoalveolar lavage fluid (BALF), pathogen detection, treatment

## Abstract

**Background:**

Community-acquired pneumonia (CAP) remains a significant cause of pediatric morbidity and mortality worldwide. Conventional microbial tests (CMTs) frequently fail to accurately identify pathogens, especially in cases involving co-infections or less common organisms. Targeted next-generation sequencing (tNGS) presents a promising alternative, offering comprehensive pathogen detection.

**Methods:**

A retrospective observational analysis was conducted on 206 pediatric CAP patients from July 2021 to January 2023. Bronchoalveolar lavage fluid (BALF) samples underwent simultaneous tNGS and CMTs. Clinical diagnoses based on comprehensive analysis served as the reference standard. Relative abundance thresholds were optimized to reduce false-positive detections.

**Results:**

Targeted next-generation sequencing detected pathogens in 97.0% (200/206) of cases, significantly higher than CMTs (52.9%, 109/206; *p* < 0.001). tNGS identified a broader spectrum of pathogens, substantially improving overall detection compared to CMTs (84.6% vs. 40.7%). Specifically, detection rates of viral pathogens (*p* < 0.05) and bacterial co-infections (*p* < 0.001) were significantly enhanced. The sensitivity and specificity of tNGS were 96.4 and 66.7%, respectively. Additionally, tNGS demonstrated superior diagnostic concordance with clinical diagnoses in both single and co-infection cases. Optimizing relative abundance thresholds reduced the false-positive rate from 39.7 to 29.5% (*p* < 0.0001). Clinical management was adjusted based on tNGS results in 41.7% of patients, significantly shortening hospital stays in severe CAP cases (*p* < 0.01).

**Conclusion:**

Targeted next-generation sequencing provides significantly improved pathogen detection, especially for co-infections, compared to CMTs. Implementing standardized relative abundance thresholds enhances the diagnostic specificity of tNGS, supporting its integration into routine clinical diagnostics for pediatric CAP to facilitate precise, timely therapeutic interventions.

## 1 Introduction

Community-acquired pneumonia (CAP) remains one of the leading causes of morbidity and mortality in children under 5 years of age, particularly in low-and middle-income countries (Torres et al., 2021). According to the 2021 Global Burden of Disease (GBD) report, pneumonia is responsible for 13.3% of all deaths in this age group, underscoring its significant public health burden (GBD 2019 Under-5 Mortality Collaborators, 2021). Although the introduction of vaccines against *Haemophilus influenzae type b (Hib)* and *Streptococcus pneumoniae* has led to a decline in the incidence of bacterial pneumonia, it has also shifted the epidemiology of CAP pathogens (Meyer Sauteur, 2024). Historically, bacterial pathogens were considered the primary causes of CAP; however, recent studies indicate a growing prevalence of viral pathogens (Rueda et al., 2022; von Mollendorf et al., 2022). A multinational study across 7 countries reported that viral pathogens were responsible for 61.4% of pediatric CAP cases, while the detection rate of *S. pneumoniae* decreased to just 6.7% (Pneumonia Etiology Research for Child Health (PERCH) Study Group, 2019).

Moreover, co-infections are common in pediatric CAP, with *respiratory syncytial virus (RSV)* and *human rhinovirus*, as well as *RSV* and *S. pneumoniae*, among the most frequent pathogen combinations (Liu et al., 2023; Lv et al., 2024). The increasing complexity of co-infections presents a challenge for accurate pathogen identification. The traditional diagnostic methods for CAP, including bacterial culture, antigen detection, and polymerase chain reaction (PCR), have several limitations, particularly in the identification of co-infections and less common pathogens (Rodrigues and Groves, 2018). Culturing is time-consuming and often yields false-negative results due to prior antibiotic use. Antigen detection is limited to known pathogens, while PCR, although more sensitive, typically targets predefined markers, thus limiting its ability to detect unexpected or rare pathogens (Zar et al., 2017).

The tNGS has emerged as a promising diagnostic tool for detecting pathogens in CAP (Chen et al., 2024; Zhang et al., 2024). tNGS enables the simultaneous identification of bacteria, viruses, fungi, and other pathogens, offering superior sensitivity and specificity, particularly for co-infections (Poulsen et al., 2023; He et al., 2025). Compared to CMTs, tNGS has demonstrated higher positive detection rates and shorter turnaround times (Huang et al., 2023; Li et al., 2025). However, its clinical application is challenged by the absence of standardized interpretation criteria. Distinguishing true pathogens from commensal microbiota, transient colonizers, and background contamination remains difficult, which can lead to increased false-positive results and complicate clinical decision-making (Diao et al., 2021).

To improve result interpretation, recent studies have explored quantitative and semi-quantitative into tNGS approaches, including relative abundance thresholds (Yin et al., 2024). In clinical practice, the logarithm of reads per kilobase per million mapped reads [lg (RPKM)], genomic coverage, and relative abundance are often used to help differentiate infection-related pathogens from background noise (Liu et al., 2022). A recent study developed a Linear discriminant analysis effect size (LEfSe) analysis to distinguish the discriminant bacteria from lower respiratory tract infections (Dong et al., 2024). However, the optimal approach for defining positivity remains unclear.

This study aims to improve the diagnostic application of tNGS in pediatric CAP by evaluating its diagnostic performance against CMTs. By integrating pathogen-specific diagnostic thresholds, we seek to improve result interpretation and reduce false-positive rates, ultimately enhancing the clinical utility of tNGS in pediatric pneumonia management.

## 2 Materials and methods

### 2.1 Ethics statement

This study was approved by the institutional ethics committee of Dongguan Maternal and Child Health Hospital. All procedures complied with the ethical standards of the Commission Responsible for Human Experimentation (both institutional and national) and the 1975 Declaration of Helsinki, revised in 2000. The bronchoscopy procedure was conducted with the informed consent of all subjects. The informed consent clearly stated the purpose, necessity, and potential risks of the procedure, including possible bleeding and infection risks, as required by ethical guidelines. Additionally, for patients who underwent bronchoalveolar lavage testing, including targeted next-generation sequencing (tNGS), a specific informed consent was also obtained. Since this study was a retrospective analysis using anonymized patient records without any additional interventions or sample collection, the institutional ethics committee approved a waiver of informed consent for the use of data in this research.

### 2.2 Study design and population

This retrospective observational study enrolled children diagnosed with pneumonia and hospitalized at Dongguan Maternal and Child Health Hospital from July 2021 to January 2023. The inclusion criteria included: (i) children diagnosed with pneumonia; (ii) aged between 1 month and 18 years; (iii) met the indications for bronchoscopy and successfully underwent bronchoalveolar lavage fluid (BALF) collection. Exclusion criteria were: (i) incomplete clinical data; (ii) refusal to undergo tNGS. A total of 206 children were enrolled. Data on patient age, sex, symptoms, laboratory findings, lung imaging, bronchoscopic findings, and medical history were recorded. All patients underwent bronchoscopy to obtain BALF samples for CMTs and tNGS analysis. Bronchoscopies were conducted by experienced physicians following standard safety protocols, with no serious adverse events reported.

### 2.3 Samples collection and storage

Bronchoscopy was performed using a flexible fiberoptic bronchoscope (Olympus) to collect three of 3–5 mL of BALF. Two of the samples were designated for culture and PCR analysis, the third sample immediately cooled and stored at 4°C to preserve the integrity of nucleic acids until further processing.

### 2.4 Conventional microbial tests

Routine samples, including BALF, sputum, and blood, were collected. CMTs conducted within average 3 days of admission included blood, sputum, and BALF cultures and smears (acid-fast staining for *Mycobacterium tuberculosis*), nasopharyngeal (NP) swab multiplex PCR for pathogens such as *Respiratory syncytial virus, Adenovirus, Rhinovirus, Mycoplasma pneumoniae, Influenza A virus, Influenza B virus*, BALF PCR for *M. pneumoniae* and *Cytomegalovirus*, and serum antibody tests for *M. pneumoniae, Cytomegalovirus*, and *Epstein–Barr viruses*.

### 2.5 tNGS

#### 2.5.1 Sample preparation

All tNGS analyses were performed on BALF specimens only. Other specimen types were not included in the tNGS analysis. A volume of 650 μL of the sample was mixed with an equal volume of 80 mmol/L dithiothreitol (DTT) in a 1.5 mL centrifuge tube and vortexed thoroughly for 10 s. Throughout the experiment, positive and negative controls from the Respiratory Pathogen Detection Kit (KS608-100HXD96, KingCreate, Guangzhou, China) were used to monitor the entire targeted NGS process.

#### 2.5.2 Nucleic acid extraction

A volume of 250 μL of the homogenized sample was used for nucleic acid extraction and purification following the manufacturer’s protocol for the Proteinase K lyophilized powder (R6672B-F-96/48/24, Magen, Guangzhou, China) to obtain high-quality total nucleic acid.

#### 2.5.3 Library construction and sequencing

Library construction was performed using the Respiratory Pathogen Detection Kit, with a no-template control set up to monitor the library construction and sequencing process. The library construction involved two rounds of PCR amplification, where the sample nucleic acid and cDNA were used as templates. A set of 153 microorganism-specific primers was used for ultra-multiplex PCR amplification to enrich the target pathogen sequences, including bacteria, viruses, fungi, mycoplasma, and chlamydia, which covered for more than 95% of the respiratory infection (Li et al., 2022). The amplified PCR products were purified using magnetic beads and subsequently re-amplified using primers containing sequencing adapters and unique barcodes. The quality and quantity of the constructed library were assessed using the Qsep100 Bio-Fragment Analyzer (Bioptic, Taiwan, China) and the Qubit 4.0 fluorometer (Thermo Scientific, Massachusetts, United States). Typically, the library fragments were within the size range of 250 to 350 bp, and the library concentration was maintained at a minimum of 0.5 ng/μL. The concentration of the pooled library was reassessed and diluted to a final concentration of 1 nmol/L. Subsequently, 5 μL of the pooled library was mixed with 5 μL of freshly prepared 0.1 mol/L NaOH, vortexed briefly, centrifuged, and incubated at room temperature for 5 min. The diluted and denatured library was then subjected to sequencing on an Illumina MiniSeq platform using a universal sequencing reagent kit (KS107-CXR, KingCreate, Guangzhou, China). On average, each library yielded approximately 0.1 million reads with a single-end read length of 100 bp.

### 2.6 Bioinformatics analysis

Sequencing data were analyzed using the data management and analysis system (v3.7.2, KingCreate). First, raw data were initially identified, retaining single-end reads exceeding 50 bp, followed by low-quality filtering to keep reads with Q30 > 75% for high-quality data. The single-end aligned reads were then compared against the self-constructed clinical pathogen database to determine the read count of specific amplification targets in each sample. The reference sequences used for read mapping were sourced from multiple databases, including the GenBank, RefSeq, and Nucleotide databases from NCBI.

### 2.7 Interpretation of tNGS results

Based on the principle of targeted amplification of microbial sequences using specific primers, the amplicon coverage and normalized read count of detected microorganisms were the primary indicators for result interpretation. The criteria for categorizing a microorganism as a positive result were as follows: (i) bacteria (excluding the *M. tuberculosis complex*), fungi, and atypical pathogens: amplicon coverage ≥50% and normalized read count ≥10; (ii) viruses: amplicon coverage ≥50% and normalized read count ≥3, or normalized read count ≥10; (iii) *M. tuberculosis complex*: normalized read count ≥1.

### 2.8 tNGS pathogen detection criteria using relative abundance

Relative abundance (RA) was introduced as a metric for pathogen detection in tNGS results. RA is calculated by dividing the normalized read count of a specific pathogen by the total read count of all detected pathogens in a sample. This standardizes the results across different samples, reducing biases caused by differences in sequencing depth or pathogen load. As multiple pathogens may be detected in the same sample, not all detected microorganisms are true pathogens. To minimize false positives, pathogen-specific RA thresholds were determined using the maximum Youden index. Pathogens with RA values above this threshold are classified as potential pathogens, while those below the threshold are considered background microorganisms.

However, discrepancies were observed between bioinformatics results and clinical presentation. For example, *Tropheryma whipplei* and *Pneumocystis jirovecii* were initially detected as potential pathogens based on their RA values, but clinicians determined that these microorganisms were not responsible for the clinical symptoms. Consequently, these microorganisms were reclassified as background organisms, rather than true pathogens.

To further validate tNGS results, patients were categorized according to their tNGS outcomes and final clinical diagnoses as follows: True-positive (TP) — patients with positive tNGS results and a confirmed diagnosis of pulmonary infection; True-negative (TN) — patients with negative tNGS results and a final diagnosis of no pulmonary infection; False-negative (FN) — patients with negative tNGS results but a confirmed pulmonary infection; False-positive (FP) — patients with positive tNGS results but no evidence of a pulmonary infection.

### 2.9 Clinical comprehensive analysis as reference standard

Two experienced clinicians reviewed all patients’ clinical data, including CMTs and tNGS results, along with their medical histories. Initially, each clinician assessed whether the patient met the diagnostic criteria for pneumonia, as outlined by the government guideline (National Health Commission of the People’s Republic of China and State Administration of Traditional Chinese Medicine, 2019). This assessment included consideration of clinical symptoms, pulmonary imaging, and laboratory findings. Following this, the etiology was established through a comprehensive review of the patient’s clinical presentation, laboratory results, imaging, microbiological tests, and treatment response. In cases of disagreement, a senior clinician was consulted to reach a consensus, ensuring the accuracy and consistency of the clinical diagnosis.

### 2.10 Statistical analysis

SPSS 26 (IBM Corporation) was utilized for all analyses. Clinical diagnosis and microbiological etiology determination served as reference standards. Pathogen analysis involved calculating sensitivity, specificity, positive predictive value, negative predictive value, and accuracy using standard proportion formulas. Wilson’s method determined 95% confidence intervals for these measures. McNemar’s test compared the diagnostic performances of CMTs and tNGS. All tests were two-tailed, with a *p*-value of <0.05 deemed statistically significant. Some children with multiple microbial infections were assigned multiple class labels (bacteria, viruses, fungi, and atypical pathogens) in this study. Sensitivity, specificity, accuracy, and positive predictive value are reported as performance metrics to facilitate direct comparisons between tNGS and CMTs.

## 3 Results

### 3.1 Patient characteristics

**Figure 1:**
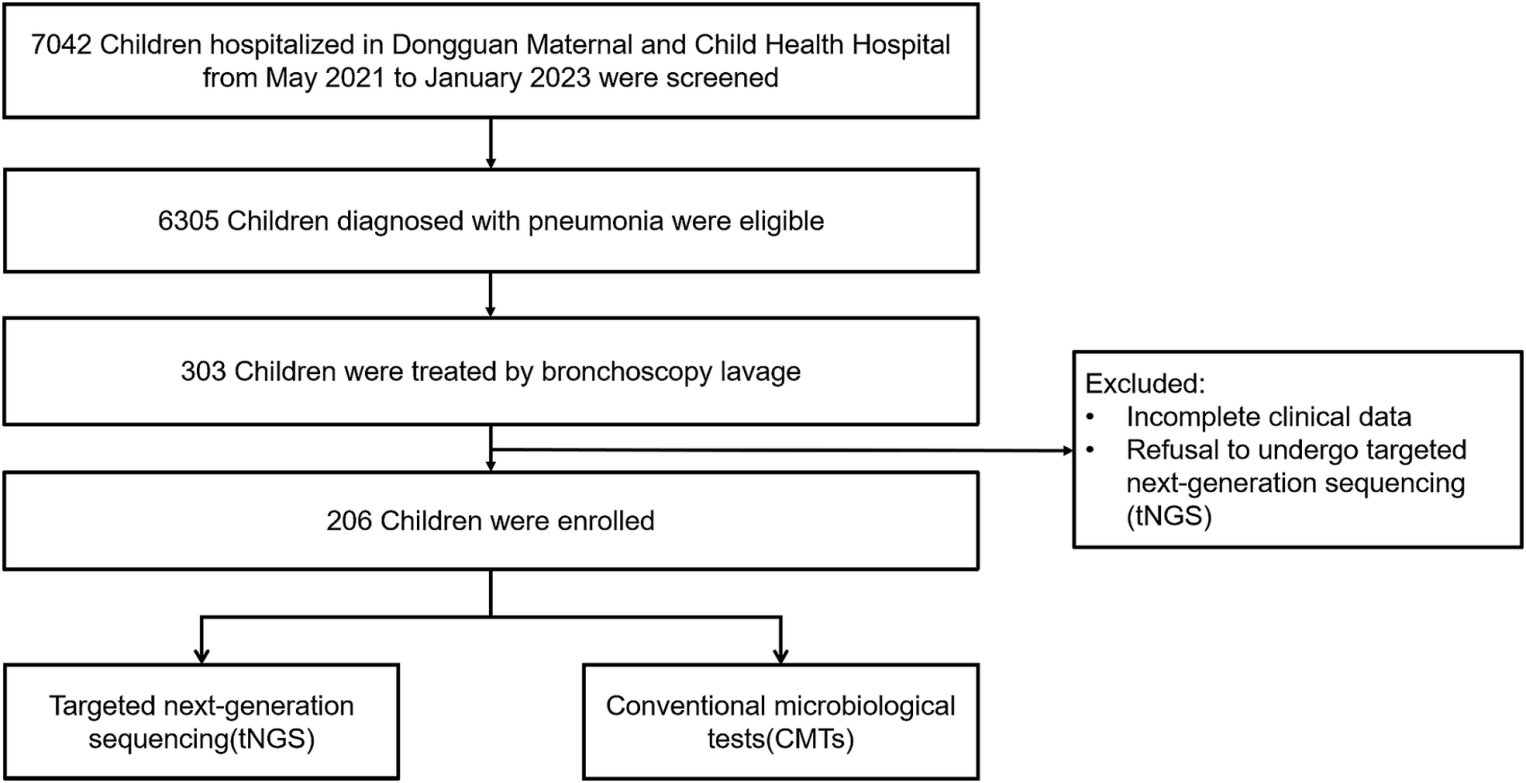
The recruitment process of the study.

Among the 7,042 children with CAP who were eligible for screening, 303 (4.3%) met the criteria for enrolment. Ninety-seven patients were excluded due to missing data and refusal to undergo tNGS, and finally, 206 patients met the inclusion criteria (Figure 1). The median age of CAP patients was 24 months (7, 48 months). Of these, 129 (62.6%) were male. Twenty-two patients were assessed as high-risk because of airway developmental abnormalities, genetic diseases, or severe growth retardation. Cough, fever, wheezing, and the tri-retraction sign were observed in 206 (100%), 151 (73.3%), 76 (36.9%), and 57 (27.7%) patients on admission, respectively, which were the most common symptoms. The median temperature on admission was 37.1°C (36.6, 38). The median hospital stay for the patients was 7.5 days (6, 10 days), and BALF samples were collected 2 days (1, 4 days) after admission. Consolidation was found in 25 (12.1%) patients, emphysema or atelectasis in 23 (11.2%) patients, and pleural effusion in 6 (2.9%) patients. Eighty-six (41.7%) patients were diagnosed with severe pneumonia, and 23 (11.2%) required mechanical ventilation for an average of 4.1 days. Further clinical details, including demographic data, laboratory results, and prognostic information, are presented in Table 1.

**Table 1:** Clinical characteristics of children.

Characteristics	Patients (*n* = 206)
Age, months	24(1–179)
Sex, male	129 (62.6%)
Symptoms or signs	
Cough	206 (100%)
Fever	151 (73.3%)
Whoop	76 (36.9%)
Tri-retraction sign	57 (27.7%)
Temperature (°C)	37.4 ± 0.9
Heart rate	133 ± 20
Respiratory	40 ± 12
Chest radiograph	
Consolidation	25 (12.1%)
Emphysema	14 (6.8%)
Atelectasis	9 (4.4%)
Pleural effusion	6 (2.9%)
Interstitial changes	2 (1.0%)
Laboratory parameters	
White Blood Cell Counts (×10^9/L)	10.5 ± 5.3
Neutrophil (%)	47.2 ± 19.6
Lymphocyte (%)	41.5 ± 17.9
Lactate dehydrogenase (U/L)	305.4 ± 93.9
C-reactive protein (mg/L)	27.0 ± 45.6
Procalcitonin (ng/ml)	1.03 ± 3.92
Severe pneumonia	86 (41.7%)
Mechanical ventilation	23 (11.2%)

### 3.2 Optimization of tNGS thresholds and enhanced pathogen detection in pediatric pneumonia

Using clinical diagnosis as the gold standard, we analyzed the tNGS results by incrementally increasing each pathogen’s relative abundance threshold in 0.1% steps and observing changes in sensitivity and specificity. The optimal threshold for each pathogen was defined by the maximum Youden’s index (sensitivity + specificity – 100%). At this optimal threshold, the corresponding sensitivity and specificity varied for each pathogen. Accordingly, 0.0% was an appropriate threshold for *H. influenzae*, *S. pneumoniae*, *M. pneumoniae*, and *Respiratory syncytial virus (RSV)*, 1.0% for *Chlamydia trachomatis* and *M. tuberculosis complex*, 2.0% for *Human metapneumovirus*, 63% for *Pseudomonas aeruginosa*, 76.0% for *Human coronavirus*, and 83.0% for *Human bocavirus type 1* (Table 2).

**Table 2:** Threshold of pathogens detection result by tNGS testing.

Pathogen species	Threshold (RA)
Atypical bacteria	
*Mycoplasma pneumoniae* + A3	0.0%
*Bordetella pertussis*	0.0%
*Chlamydia pneumoniae*	0.0%
*Chlamydia trachomatis*	1.0%
*Legionella pneumophila*	NA
Bacteria	
*Haemophilus influenzae*	0.0%
*Moraxella catarrhalis*	0.0%
*Staphylococcus aureus*	0.0%
*Streptococcus pneumoniae*	0.0%
*Acinetobacter baumannii*	0.0%
*Escherichia coli*	0.0%
*Klebsiella pneumoniae*	0.0%
*Mycobacterium tuberculosis* complex	1.0%
*Pseudomonas aeruginosa*	63.0%
Viruses	
Cytomegalovirus	0.0%
Respiratory syncytial virus	0.0%
Rhinovirus	0.0%
Adenovirus	0.0%
Enterovirus D68	0.0%
Enterovirus group B	0.0%
Epstein–Barr virus	0.0%
Human bocavirus type 1	83.0%
Human coronavirus	76.0%
Human metapneumovirus	2.0%
Human parainfluenza virus	0.0%
Influenza B virus	0.0%

The optimal threshold for *P. aeruginosa* was much higher than that for other bacteria, possibly because infections with *P. aeruginosa* are often chronic and occur in patients with underlying immune deficiencies. Meanwhile, the optimal thresholds for *Human coronavirus* and *Human bocavirus type 1* were extremely high, which might be related to their atypical clinical presentations—only when the relative abundance is very high (indicating severe infection) are these viruses considered to be in an active infectious phase.

**Figure 2:**
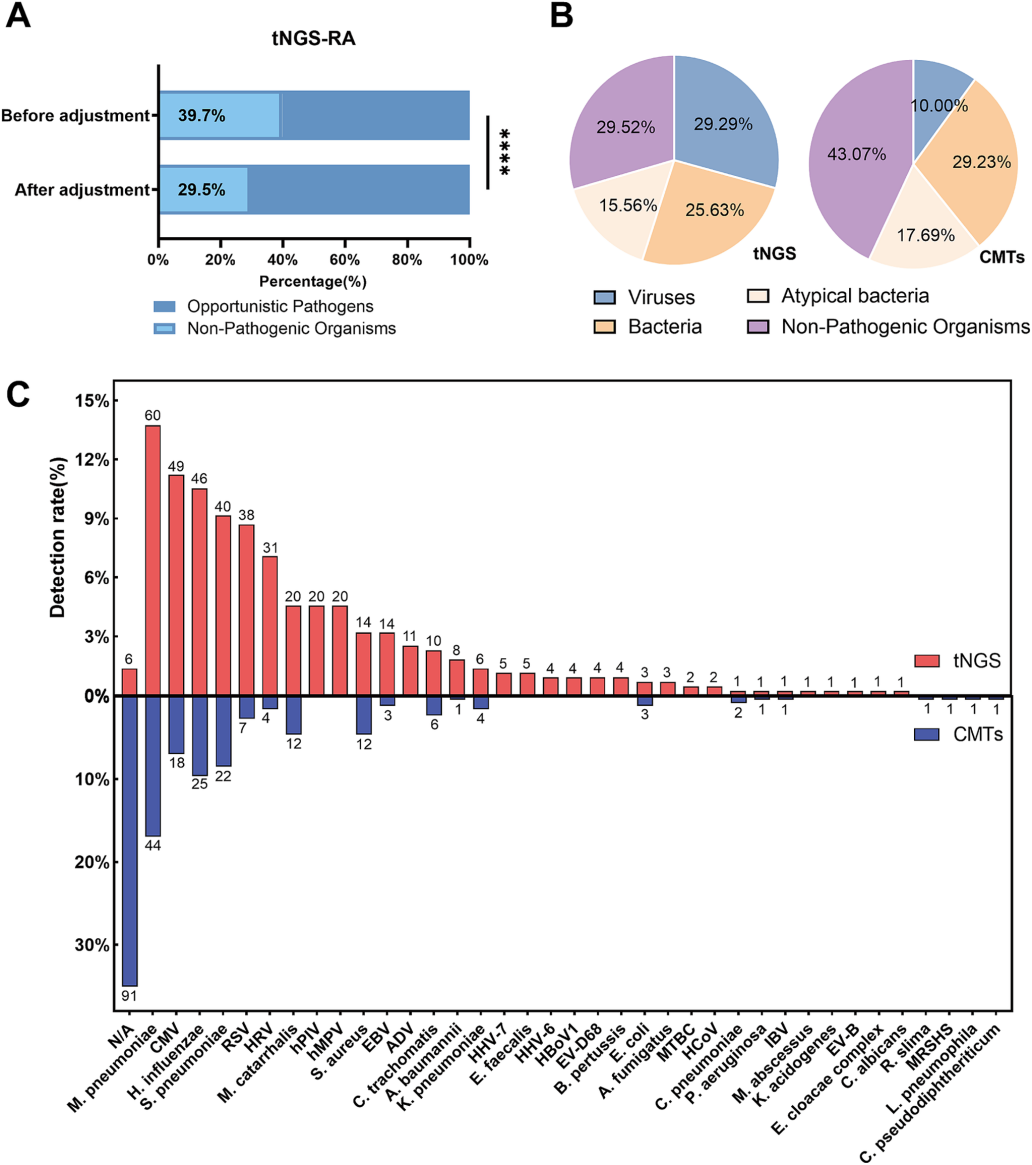
Microorganisms detection in pediatric Community-Acquired Pneumonia using tNGS and CMTs. **(A)** The bar chart illustrates the relative abundance of pathogens identified by tNGS before and after data adjustment. **(B)** Pie charts depict the distribution of detected microorganisms, categorized into viruses, bacteria, atypical bacteria, and non-pathogenic organisms by both tNGS and CMTs. **(C)** This figure illustrates the detection rates of microorganisms detected by tNGS and CMTs (shown above the bars). tNGS identified a total of 35 microorganisms in 200 patients, while CMTs detected 20 microorganisms in 109 patients.

Based on the tNGS results for 206 patients, a total of 511 microorganisms’ occurrences were initially detected. After applying the pathogen-specific optimal thresholds, the number of positive detections was reduced to 437, thereby lowering the tNGS false-positive rate from 39.7 to 29.5% (*p* < 0.0001, Figure 2A). Compared with CMTs, tNGS yielded a significantly higher detection rate of pathogenic microorganisms, whereas the majority of pathogens detected by CMTs were bacteria (Figure 2B).

In this study, tNGS identified 35 microorganisms across 200 patients (97.0%), including 15 bacteria, 14 viruses, 4 atypical bacteria, and 2 fungi. In comparison, conventional microbial tests detected 20 different microorganisms in 109 patients (52.9%), including 13 bacteria, 4 viruses, and 3 other prokaryotes. The most frequently identified microorganisms by tNGS were *M. pneumoniae* (12.4%), *Cytomegalovirus* (10.1%), and *H. influenzae* (9.5%). For conventional microbial tests, *M. pneumoniae* (26.0%), *H. influenzae* (14.8%), and *S. pneumoniae* (13.0%) were the most common microorganisms detected (Figure 2C).

### 3.3 Pathogen detection performance of tNGS and CMTs

**Figure 3:**
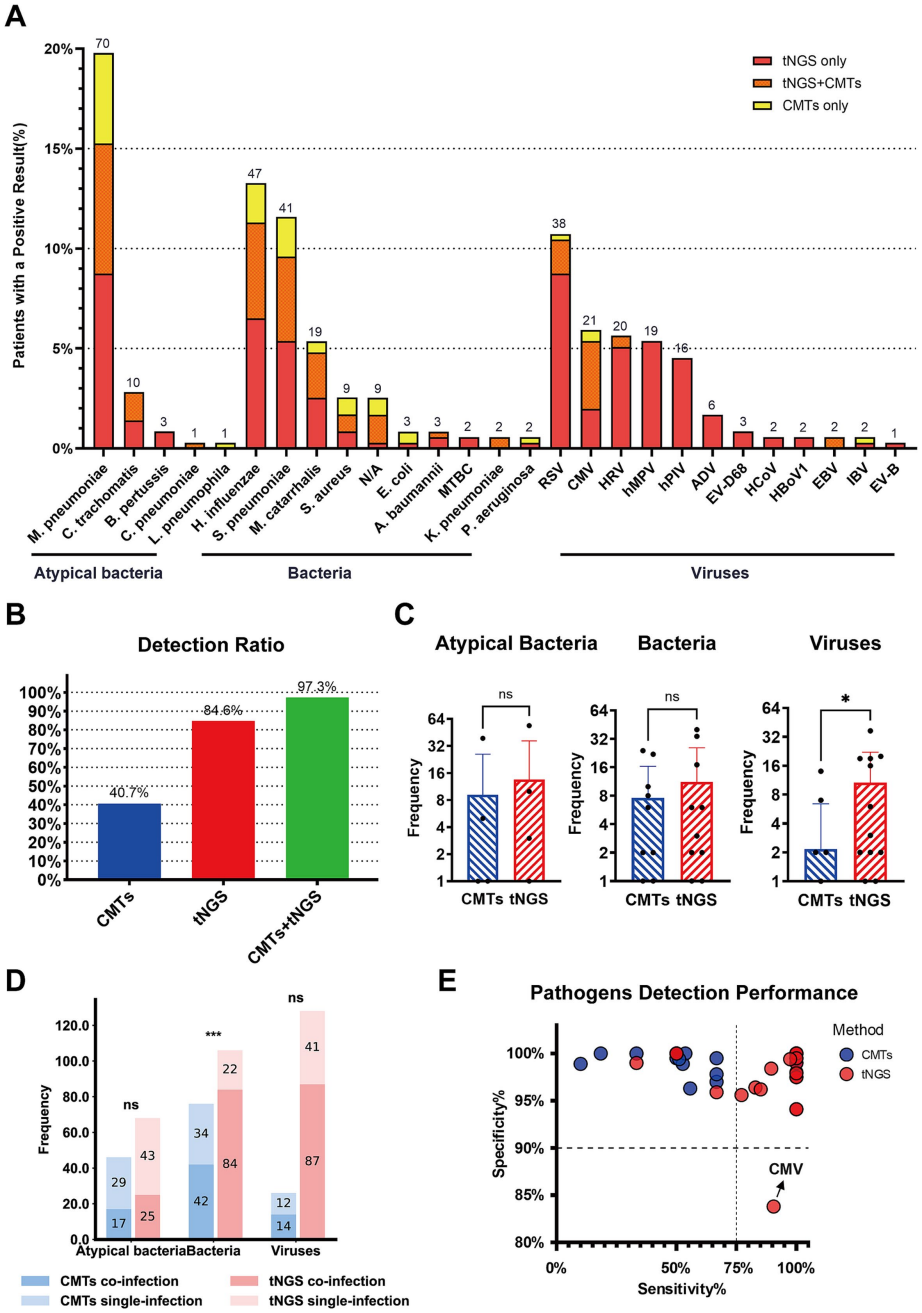
Pathogen identification in Pediatric Community-Acquired Pneumonia using tNGS and CMTs. **(A)** The stacked bar chart shows the numbers (above the bars) and percentages (*y*-axis) of specific pathogens detected. A total of 354 pathogens were detected in 206 patients, of which 197 patients got positive etiology results. **(B)** The bar chart on the left shows the pathogen detection ratio with different detection methods combinations. **(C)** The bar charts compare the frequency of detection of atypical bacteria, bacteria, and viruses by tNGS and CMTs. The *y*-axis indicates the frequency of detections, and the results show no significant difference (ns) in the detection of atypical bacteria and bacteria between the two methods, while there is a significant difference (*) in virus detection. **(D)** This bar chart shows the frequency of pathogens detected in single and co-infections, comparing tNGS and CMTs across atypical bacteria, bacteria, and viruses. The results show no significant difference (ns) in the detection of atypical bacteria and viruses between the two methods, while there is a significant difference (***) in bacteria detection. **(E)** The bubble chart represents the sensitivity and specificity of specific pathogens detected by tNGS and CMTs.

Among the 206 children with pneumonia (including both single and co-infections), a total of 364 pathogens were clinically identified. Of these, 354 pathogens were detected using both tNGS and CMTs. However, 10 pathogens (bacteria), although clinically confirmed, were not identified by either method. The most common pathogens included *M. pneumoniae* (70/354, 19.8%), *H. influenzae* (47/354, 13.3%), *S. pneumoniae* (41/354, 11.6%), and *Respiratory syncytial virus* (38/354, 10.7%). Other frequently pathogens included *Cytomegalovirus* (21/354, 5.9%), *Rhinovirus* (20/354, 5.6%), *Moraxella catarrhalis* (19/354, 5.4%), *Human metapneumovirus* (19/354, 5.4%), and *Parainfluenza virus* (16/354, 4.5%) (Figure 3A).

The positive detection rate using tNGS was 84.6% (308/364), compared to only 40.7% (148/364) with CMTs. Combining CMTs with tNGS significantly increased the detection rate to 97.3% (354/364) (Figure 3B). tNGS demonstrated higher positive rates for detecting viruses, bacteria, and atypical pathogens than CMTs, with a significant difference in the detection rates of viral pathogens (*p* < 0.05). Although tNGS also showed higher detection rates for bacteria and atypical pathogens, these differences were not statistically significant (Figure 3C). Furthermore, tNGS outperformed CMTs in the detection of bacterial co-infections, emphasizing its advantage in diagnosing complex infection cases (Figure 3D).

In general, tNGS showed higher sensitivity than CMTs for bacterial and atypical pathogens while maintaining similar specificity. For *M. pneumoniae*, tNGS exhibited both higher sensitivity and similar specificity. However, for *CMV*, the specificity of tNGS (83.8%) is significantly lower than that of CMTs (97.8%) (Figure 3E), possibly due to non-specific amplification when detecting low-abundance or clinically irrelevant viral DNA fragments. A detailed comparison of diagnostic metrics—including sensitivity and specificity—for different pathogen categories is provided in Supplementary Table 1.

### 3.4 Diagnostic performance of tNGS and CMTs in individual pediatric patients

**Figure 4:**
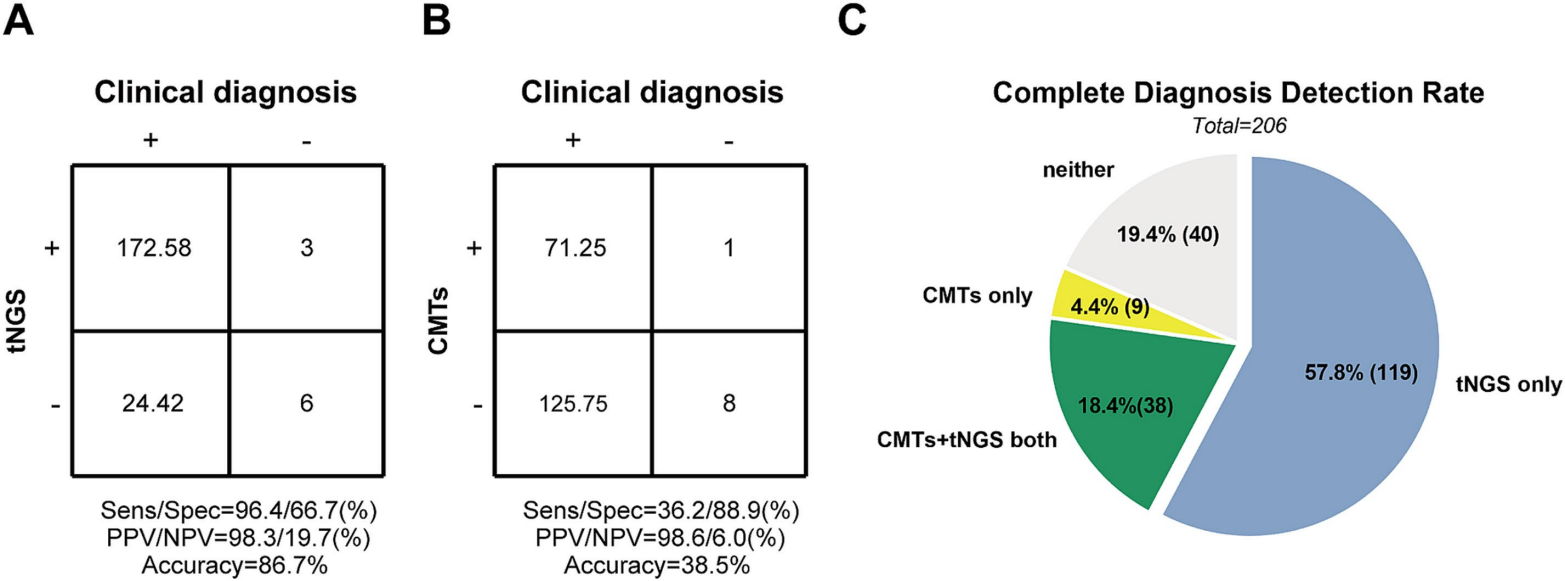
The diagnosis performance of tNGS and CMTs in Pediatric Community-Acquired Pneumonia patients. **(A,B)** Accuracy evaluation of tNGS and CMTs by 2 × 2 contingency tables based on the Clinical diagnosis, respectively. **(C)** Comparison of diagnosis performance between tNGS and CMTs. The pie chart shows the distribution of diagnosis consistency between tNGS and CMTs methods.

Among the 206 enrolled patients, 9 (4.4%) had negative pathogen results, while 197 patients had positive pathogen results. Based on clinical diagnosis, 197 samples tested positive 97.0% of the time (191/197) using tNGS, with complete pathogen agreement in 76.6% of cases (151/197). Partial consistency was observed in 40 samples, indicating the presence of at least one pathogen in mixed cultures. Only 6 samples (3.0%) showed inconsistent results. tNGS results scored 172.58 out of 197, indicating a sensitivity of 96.4% and a PPV of 98.3% (Figure 4A). Among the 9 diagnosis-negative samples, 6 (66.7%) were consistent with tNGS results, while 3 samples detected positive pathogens, resulting in a specificity of 66.7% and an NPV of 19.7% (Figure 4A).

For diagnosis-positive samples, CMTs detected 19.8% of pathogens identified by clinical diagnosis (39/197), while 48.7% were missed. A false-positive sample increased the CMTs score by 1.0. True-negative samples scored 8. Sensitivity and specificity were calculated to be 36.2 and 88.9%, respectively, with PPV and NPV at 98.6 and 6.0%, respectively (Figure 4B). Detailed data supporting these findings are provided in Supplementary Table 2.

Among all enrolled children with pneumonia, the detection rate of a complete diagnosis by both tNGS and CMTs combined was only 18.4% (38/206). One hundred nineteen children (57.8%) were diagnosed solely by tNGS, while only 9 (4.4%) were diagnosed solely by CMTs. Additionally, 40 cases (19.4%) were missed by both methods (Figure 4C).

### 3.5 Age-related pathogen profiles and diagnostic performance of tNGS and CMTs in single and co-infections

**Figure 5:**
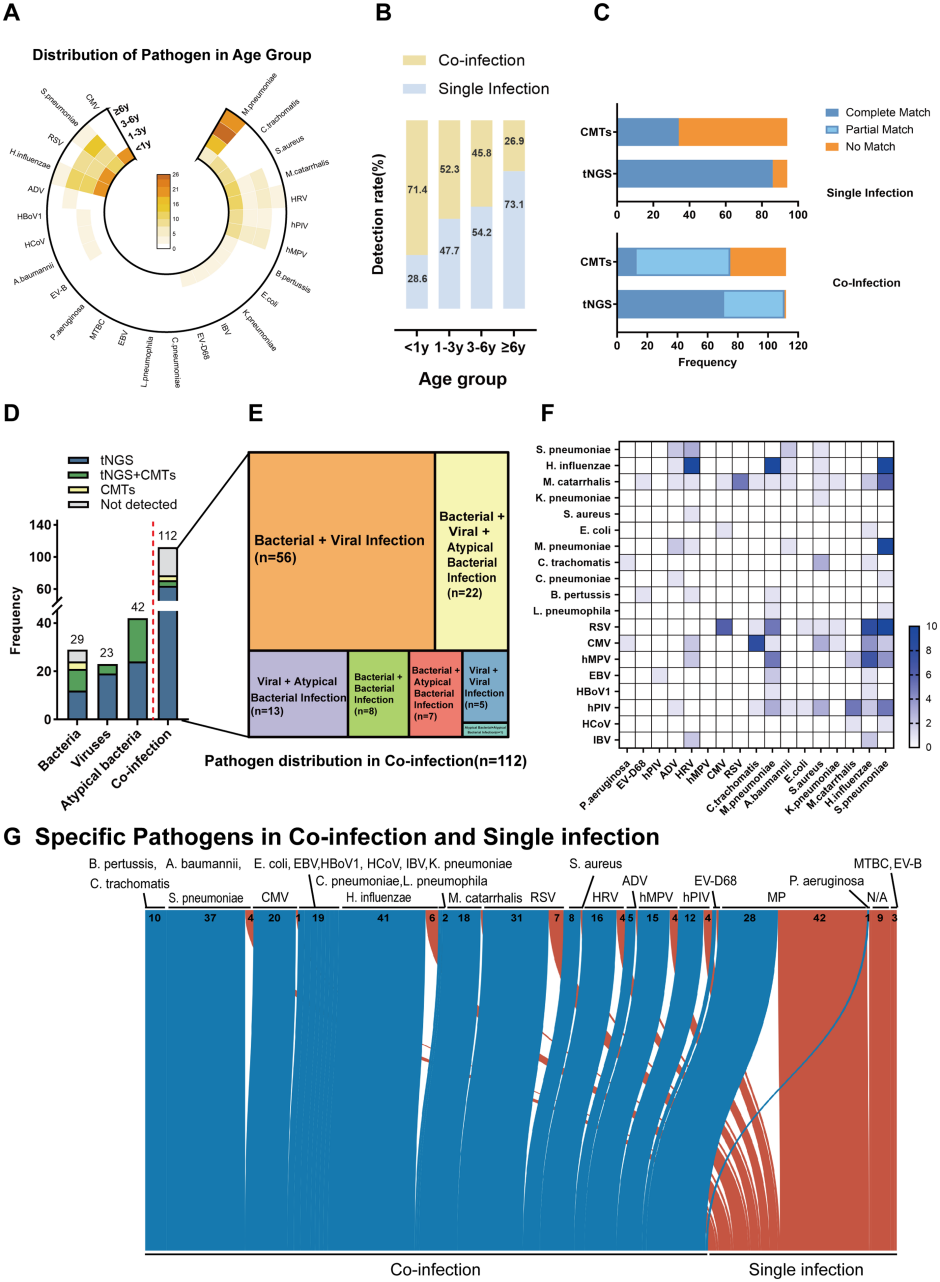
Age-dependent pathogen distribution and co-infection patterns in pediatric pneumonia detected by tNGS and CMTs. **(A)** Distribution of pathogens across age groups. **(B)** Co-infection and single infection rates by age group. **(C)** Comparison of pathogen detection results between tNGS and CMTs in single and co-infections. The *x*-axis indicates the detection methods (tNGS and CMTs), and the *y*-axis represents the frequency of detection outcomes: complete matches (blue), partial matches (light blue), and no matches (orange). **(D)** Frequency of pathogen detection in various categories including bacteria, viruses, atypical bacteria, and co-infections. The bar graph displays the counts of pathogens detected exclusively by tNGS (blue), exclusively by CMTs (green), by both methods (yellow), and not detected (gray). **(E)** Diagram representing the specific types of co-infections diagnosed in patients, where the area of each segment is proportional to the number of patients with each type of co-infection. **(F)** Heatmap illustrating the frequency of specific pathogens detected across different infection types. **(G)** Sankey diagram depicting the distribution of pathogens in co-and single infections as detected by tNGS. The width of the arrows is proportional to the number of pathogen detections, with co-infections shown in blue and single infections in red.

As shown in Figure 5A, pathogen distribution varied across age groups. Infants under 1 year were predominantly affected by viral infections, particularly *respiratory syncytial virus (RSV)* and *cytomegalovirus (CMV)*. In contrast, atypical bacteria, especially *Mycoplasma pneumoniae*, were more common in children aged ≥3 years (Supplementary Figure 1A). The detection rate of tNGS also varied by age. In infants, tNGS detected viral pathogens in 90.5–100% of cases, whereas CMTs achieved detection rates of only 18.2–66.7%. Among children aged over 3 years, tNGS identified *M. pneumoniae* in 84.8% of cases, compared to 47.8% by CMTs. For other bacterial pathogens such as *S. pneumoniae, H. influenzae*, and *M. catarrhalis*, tNGS detection rates ranged from 84.2 to 100%, higher than those of CMTs (33.3–47.4%, Supplementary Figure 1B). These results demonstrate the superior sensitivity of tNGS across a broad age range and its particular advantage in detecting viral infections in infants and atypical bacteria in older children.

With increasing age, co-infections decreased and single infections became more common (Figure 5B). Among children under 1 year of age, 71.4% (55/77) were diagnosed with co-infections. Of viral detections, 21.8% (12/55) included more than one virus and 70.9% (39/55) included a concomitant bacterial detection. In contrast, 73.1% (19/26) of children aged ≥6 years had single infections, with *M. pneumoniae* being the most commonly identified pathogen (78.9%, 15/19).

In the single-infection group, the complete match rate of tNGS was 71.7%, compared to 28.3% for CMTs. The unmatched rate was 11.8% for tNGS, significantly lower than 88.2% for CMTs. Neither method showed partial matches in this group. In co-infection cases, tNGS again outperformed CMTs, with a higher complete match rate (63.4% vs. 11.6%) and lower unmatched rate (0.9% vs. 33.0%). The partial match rate was 35.7% for tNGS, slightly below that of CMTs (55.4%) (Figure 5C). Together, these results suggest that tNGS provides more accurate identification across different infection types, particularly in reducing diagnostic failures.

Among 29 bacterial single infections, tNGS results agreed with the clinical diagnosis in 51.7% (15/29) of cases when compared with traditional “gold standards” such as blood culture, sputum culture, and bronchoalveolar lavage culture in bacterial infections. In contrast, CMTs indicated that 13.8% (4/29) of positive results were both detected by tNGS. In viral and atypical bacterial infections, tNGS identified all cases, whereas CMTs yielded positive results in only 17.4% (4/23) and 42.9% (18/42) of cases, respectively.

In the 112 co-infection cases, tNGS alone detected 64 cases (57.1%), while CMTs yielded positive results in only 13 (11.6%) (Figure 5D). The most frequent combinations involved both bacterial and viral pathogens (56/112, 50.0%), with *H. influenzae + Rhinovirus*, and *S. pneumoniae + RSV* (10/112, 4.8%) being the most frequent (Figures 5E,F). The overall pathogen spectrum detected by tNGS in co-and single-infection cases was shown (Figure 5G). These results indicate that tNGS has a significantly higher sensitivity than CMTs across age groups and infection types, particularly in co-infections. These results support its clinical utility in comprehensive pathogen detection.

### 3.6 Adjusting treatment based on tNGS results

**Figure 6:**
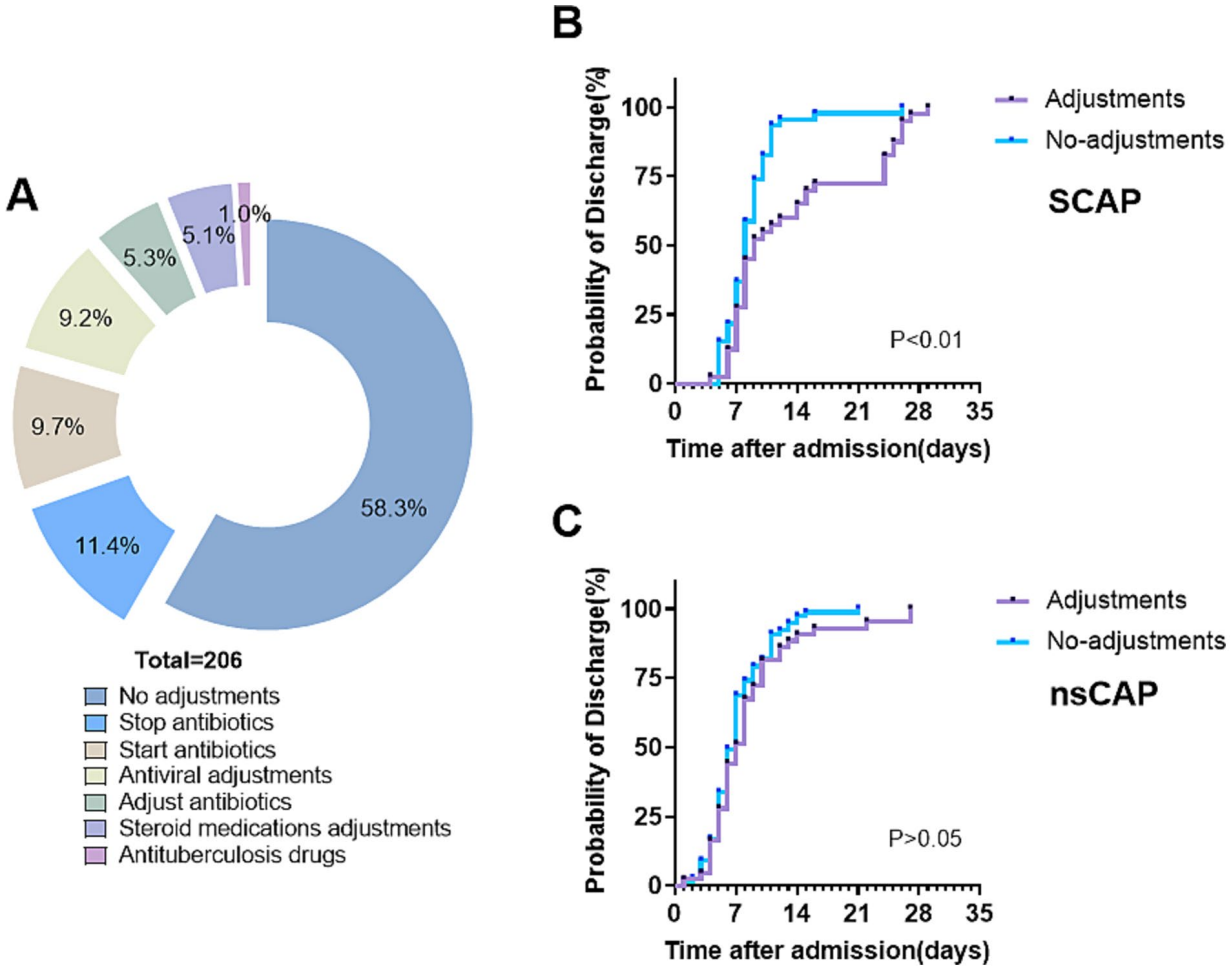
Clinical implementation of tNGS results in treatment adjustment and discharge probability. **(A)** Medication adjustments by results of tNGS. **(B,C)** Kaplan–Meier curves of the probability of discharge for patients with and without medication adjustments in severe community-acquired pneumonia (SCAP, *n* = 86) and non-severe community-acquired pneumonia (nsCAP, *n* = 120).

Based on the tNGS results, the treatment of 86 patients was adjusted, accounting for 41.7% of cases. Among these, 64 patients had their antibiotic regimens modified: 11 had their antibiotics changed, 27 had additional antibiotics prescribed, and 26 were taken off antibiotics. Additionally, 25 patients received increased antiviral treatments, and 13 received increased corticosteroid treatments (Figure 6A). After these adjustments, 98.5% of the children showed significant improvement, with 56.8% being cured. Following antibiotic adjustments, 64 patients were discharged after an average of 6.6 days. Thirteen patients, after the addition of corticosteroid treatment, were discharged after an average of 5.6 days. For the 25 patients whose antiviral drug treatments were adjusted, the average discharge time was 14.0 days, significantly longer than other groups. Patients in severe community-acquired pneumonia (SCAP) and non-severe community-acquired pneumonia (nsCAP) were divided into two groups based on medication adjustments. Discharge occurred earlier and the incidence of discharge was significantly higher in the adjustments group than in the no-adjustments group for SCAP (*p* < 0.01; Figure 6B). However, there was no significant difference between the adjustments and no-adjustments groups among nsCAP patients (*p* > 0.05; Figure 6C).

## 4 Discussion

This study retrospectively evaluated 206 pediatric community-acquired pneumonia (CAP) cases, comparing targeted next-generation sequencing (tNGS) with conventional microbiological tests (CMTs). tNGS demonstrated a broader detection range, higher positivity rates, and greater sensitivity for bacterial, viral, and atypical pathogens. Additionally, it provided enhanced detection of co-infections and exhibited strong concordance with clinical diagnoses.

Consistent with previous studies, our results confirm the superior detection capability of tNGS over conventional methods (He et al., 2025; Tan et al., 2025). The positivity rate of tNGS reached 97.0%, significantly surpassing the 52.9% positivity rate of CMTs. Additionally, tNGS detected 84.6% (308/364) of clinically confirmed pathogenic bacteria, compared to 40.7% (148/364) with CMTs. Similar findings have been reported in adult and pediatric cohorts, with tNGS consistently outperforming traditional culture-and PCR-based methods, particularly in detecting low-abundance or difficult-to-culture pathogens (Deng et al., 2023; Dai et al., 2025). This enhanced sensitivity is critical in complex infections where rapid identification of the causative agent can guide early and targeted therapy.

Our study further demonstrates that tNGS exhibits near 100.0% sensitivity and over 95.0% specificity for detecting common respiratory viruses and atypical bacteria. In bacterial infections, tNGS identified a broader spectrum and greater number of pathogens compared to culture-based methods. For instance, a previous study reported that rapid RSV diagnostic tests had a sensitivity of only 26.0%, significantly lower than the sensitivity observed with tNGS (Freeman et al., 2021). However, tNGS failed to detect certain bacteria, including *Legionella pneumophila* and *Escherichia coli*, which were identified by tNGS in previous studies (Zhang et al., 2024; Yang et al., 2025). The overall concordance between tNGS and clinical diagnoses was high, with a diagnostic sensitivity of 96.4% and a specificity of 66.7%. Compared to a study analyzing BALF samples from 47 pediatric pneumonia patients, our study found lower specificity, likely attributable to a higher false-positive rate (Lin et al., 2023; Poulsen et al., 2023). Nonetheless, when combined with rigorous quality control and clinical correlation, tNGS provides a highly accurate tool for detecting respiratory pathogens.

Despite its advantages, a major challenge of tNGS is its relatively high false-positive rate, largely due to background contamination, sequencing artifacts, or the detection of commensal microbiota. To mitigate this issue, we applied a relative abundance threshold, reducing the false-positive rate from 39.7 to 29.5% (*p* < 0.0001). Our study also provides a preliminary framework for standardizing tNGS result interpretation. A previous study has shown specific relative abundance thresholds helped differentiate pathogenic organisms from colonizers (e.g., *S. pneumoniae* at ~5%, *M. pneumoniae* at ~1%) (Chen et al., 2023). In our study, *M. pneumoniae,* which is not part of the normal microbiota, can be reported as a true pathogen regardless of abundance, whereas *P. aeruginosa* requires a threshold of 63.0% to be considered pathogenic in immunocompromised individuals. Similarly, *human bocavirus type 1* and *human coronaviruses* exhibited high optimal abundance thresholds (76.0–83.0%), suggesting that only high viral loads indicate active infection. Establishing standardized interpretation criteria will be critical for improving the clinical utility of tNGS.

The tNGS enables simultaneous detection of bacteria, viruses, fungi, and atypical pathogens, providing a comprehensive view of the microbial landscape in pediatric pneumonia. In our study, 54.4% (112/206) of cases involved co-infections. The detection rate of co-infections was significantly higher with tNGS than with CMTs (63.4%, 71/112 vs. 11.6%, 13/112), highlighting the superior sensitivity of tNGS in identifying complex infections. tNGS identified 35 distinct pathogens, with an average of two pathogens per sample, emphasizing the high prevalence of co-infections. Similar findings have been reported in large-scale studies. A study of children with severe pneumonia in Guangxi showed a high prevalence of co-infections (Tan et al., 2025). Another study on community-acquired pneumonia in children found that only 20.3% of cases involved a single pathogen, whereas nearly 80.0% had two or more co-infecting organisms, with bacterial-viral co-infections being the most common (Zhao et al., 2025). In our analysis, the prevalence of co-infection decreased with age, with younger children exhibiting a higher burden of mixed infections. Among infants under 1 year of age, 71.4% (55/77) had co-infections, and 21.8% (12/55) of these involved more than one virus. Multiple viral detections were common in this age group. Di Maio et al. (2024) reported similar findings, identifying two viruses in 24.1% and three viruses in 9.4% of pediatric pneumonia cases. In contrast, older children (over 6 years) were predominantly affected by single-pathogen infections, particularly *M. pneumoniae* (Yang et al., 2024). The ability to detect multiple pathogens in a single test provides valuable insights into disease pathogenesis and allows for more tailored antimicrobial therapy. Additionally, tNGS demonstrated superior sensitivity in detecting RNA viruses, which are often missed by conventional diagnostics (Li et al., 2022). One study found that conventional testing detected few RNA viruses, while tNGS identified them in all corresponding cases and enabled subtyping (e.g., differentiating influenza subtypes) (Wang et al., 2024). This broad-spectrum capability reinforces its potential for improving diagnostic accuracy in pediatric pneumonia.

The clinical utility of tNGS extends beyond pathogen detection to influencing treatment strategies (Deng et al., 2023). In our study, 30.3% of patients required treatment escalation based on tNGS results (e.g., additional antimicrobial coverage or a change in regimen), while 11.4% had treatment de-escalation, avoiding unnecessary antibiotics. In cases of severe CAP, targeted antimicrobial adjustments based on tNGS findings were associated with shorter hospital stays and improved clinical outcomes (*p* < 0.01). These findings suggest that early, pathogen-directed therapy guided by tNGS can optimize antimicrobial use and improve patient prognosis.

Beyond individual patient management, tNGS serves as a valuable tool for epidemiological surveillance and pathogen tracking. In our study, *respiratory syncytial virus (RSV), cytomegalovirus (CMV), rhinovirus, human metapneumovirus*, and *parainfluenza virus* were frequently associated with co-infections. We found the most common were *RSV, CMV,* and *rhinovirus*, and *CMV* emerged as the second most common viral pathogen after *RSV*, differing from previous reports (Wang et al., 2021). One possible explanation is a shift in viral epidemiology following COVID-19, as some studies suggest an increased risk of *CMV* reactivation in post-COVID cases (Zhang et al., 2023). These findings have implications for public health strategies.

This study has several limitations. It was a single-center, retrospective study with a relatively small sample size, which may limit the generalizability of the findings. The results should therefore be interpreted with caution and validated in larger, prospective, multi-center studies to ensure broader applicability. Differences in specimen types between tNGS and conventional microbiological tests may also have affected the comparability of diagnostic performance. Future studies using matched samples for both methods are warranted to enable more direct performance comparisons. The diagnostic thresholds used for pathogen identification also require further refinement and independent validation. In addition, fungal pathogens were not included, which restricted the scope of pathogen detection. Another limitation is that tNGS cannot distinguish between live and dead microorganisms. The presence of microbial DNA or RNA may reflect past infections or environmental contamination, and does not necessarily indicate active disease.

Future research should focus on improving result interpretation and diagnostic accuracy, particularly in relation to threshold settings and inclusion of a wider range of pathogens. Clinical trials are also needed to evaluate the real-world impact of tNGS-guided treatment strategies on patient outcomes. As the technology continues to advance and becomes more affordable, tNGS is expected to play an increasingly important role in the accurate and timely diagnosis of pediatric pneumonia, supporting more precise and efficient infection management.

## 5 Conclusion

Targeted next-generation sequencing (tNGS) provides significant advantages in pediatric pneumonia diagnostics by enhancing pathogen detection, identifying co-infections, and informing treatment decisions. Our study supports the clinical utility of tNGS and introduces an approach to reducing false-positive rates through optimized abundance thresholds. Further standardization and technological advancements will be essential for integrating tNGS into routine clinical practice. With continued research and cost reductions, tNGS has the potential to transform infectious disease diagnostics, improving accuracy and efficiency in pneumonia management.

## Data Availability

The data presented in this study are deposited in the Zenodo repository, DOI: 10.5281/zenodo.15664668.

## Glossary

N/A: Negative
*M. pneumoniae*: *Mycoplasma pneumoniae*
CMV: Cytomegalovirus
*H. influenzae*: *Haemophilus influenzae*
*S. pneumoniae*: *Streptococcus pneumoniae*
RSV: Respiratory syncytial virus
HRV: Human Rhinovirus
hPIV: Human parainfluenza virus
*M. catarrhalis*: *Moraxella catarrhalis*
hMPV: Human metapneumovirus
EBV: Epstein–Barr virus
*S. aureus*: *Staphylococcus aureus*
ADV: Adenovirus
*C. trachomatis*: *Chlamydia trachomatis*
*A. baumannii*: *Acinetobacter baumannii*
*K. pneumoniae*: *Klebsiella pneumoniae*
*E. faecalis*: *Enterococcus faecalis*
HHV-7: Human herpesvirus type 7
*B. pertussis*: *Bordetella pertussis*
EV-D68: Enterovirus D68
HBoV1: Human bocavirus type 1
HHV-6: Human herpesvirus type 6
*E. coli*: *Escherichia coli*
*A. fumigatus*: *Aspergillus fumigatus*
HCoV: Human coronavirus
MTBC: *Mycobacterium tuberculosis* complex
EV-B: Enterovirus group B
*C. pneumoniae*: *Chlamydia pneumoniae*
IBV: Influenza B virus
*P. aeruginosa*: *Pseudomonas aeruginosa*
*C. albicans*: *Candida albicans*
K. acidogenes: Klebsiella acidogenes
*M. abscessus*: *Mycobacterium abscesses*
*E. cloacae* complex: *Enterobacter cloacae* complex
*C. pseudodiphtheriticum*: *Corynebacterium pseudodiphtheria*
MRSHS: Methicillin-resistant hemolytic staphylococcus
*L. pneumophila*: *Legionella pneumophila*
R. slima: Ross slima
