# Supplementary material for: Comparative and clinical impact of targeted next-generation sequencing in pediatric pneumonia diagnosis and treatment
Source: Front Microbiol. 2025 Jun 25;16:1590792. doi: 10.3389/fmicb.2025.1590792 (PMC12238002; doi:10.3389/fmicb.2025.1590792)
Supplement: Supplementary file 1 [file Presentation_1.pdf]

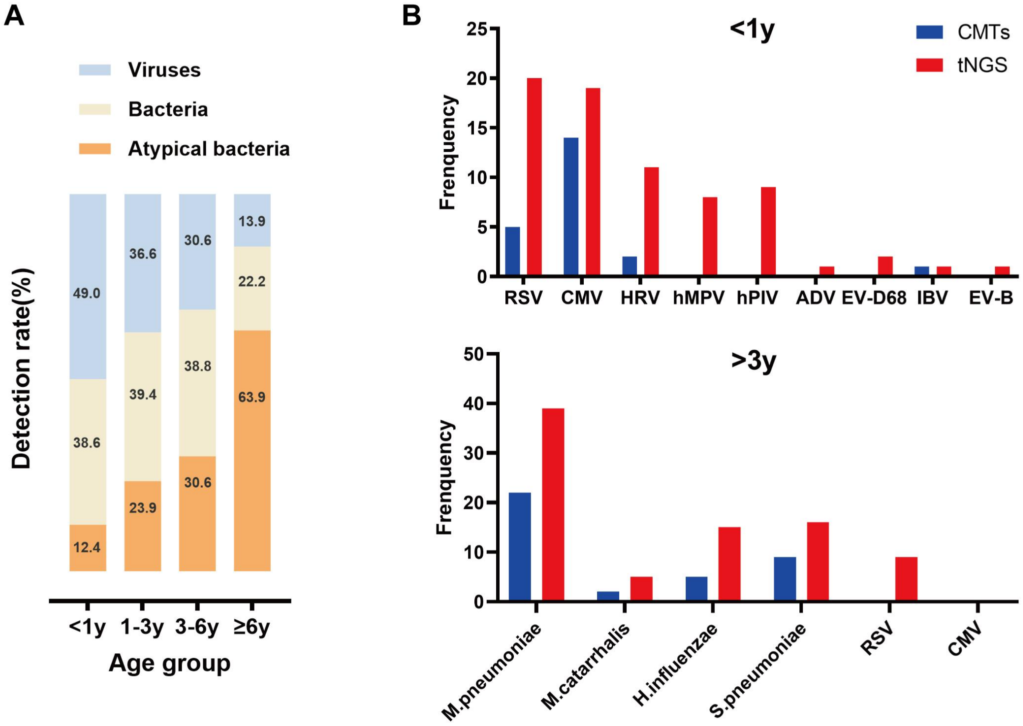


**Supplementary Figure 1.** Distribution of pathogen detection results in pediatric pneumonia across different age groups. (A) The detection rate of viruses, bacteria, and atypical bacteria across different age groups (<1 year, 1–3 years, 3–6 years, and ≥ 6 years). (B) Comparison of pathogen detection frequencies between conventional microbiological tests (CMTs, blue) and targeted next-generation sequencing (tNGS, red) in children <1 year (upper panel) and >3 years (lower panel).
